# Comparison of Traditional and Virtual Reality-Based Episodic Memory Performance in Clinical and Non-Clinical Cohorts

**DOI:** 10.3390/brainsci12081019

**Published:** 2022-07-31

**Authors:** Michael D. Barnett, Carmen J. W. Chek, Sydni S. Shorter, Thomas D. Parsons

**Affiliations:** 1 Department of Psychology and Counseling, The University of Texas at Tyler, 3900 University Blvd, Tyler, TX 75701, USA; mbarnett@uttyler.edu (M.D.B.); carmen.chek@wayne.edu (C.J.W.C.); sydni.shorter@utsouthwestern.edu (S.S.S.); 2 Grace Center, Edson College, Arizona State University, Tempe, AZ 85281, USA; 3 Computational Neuropsychology and Simulation, Arizona State University, Tempe, AZ 85281, USA

**Keywords:** virtual environment grocery store, California verbal learning test-II, virtual reality, episodic memory

## Abstract

The California Verbal Learning Test, Second Edition (CVLT-II) and the Virtual Environment Grocery Store (VEGS) use list learning and recognition tasks to assess episodic memory. This study aims to: (1) Replicate prior construct validity results among a new sample of young adults and healthy older adults; (2) Extend this work to a clinical sample of older adults with a neurocognitive diagnosis; (3) Compare CVLT-II and VEGS performance among these groups; and (4) Validate the independence of CVLT and VEGS episodic memory performance measures from executive functioning performance measures. Typically developing young adults (*n* = 53) and older adults (*n* = 85), as well as older adults with a neurocognitive diagnosis (*n* = 18), were administered the CVLT-II, VEGS, and D-KEFS CWIT. Results found that (1) the relationship of the VEGS and CVLT-II measures was highly correlated on all variables, (2) compared to the CVLT-II, participants (particularly older adults) recalled fewer items on the VEGS, and (3) the CVLT-II and VEGS were generally independent of D-KEFS CWIT. It appeared that the VEGS may be more difficult than the CVLT-II, possibly reflecting the word length effect. Performance may have also been impacted by the presence of everyday distractors in the virtual environment.

## 1. Introduction

Episodic memory refers to the storage of distinctive experiences or events associated with explicit places and times [1]. It is important for the effective performance of several activities of daily living and enables spatial and temporal recall of prior learning [2]. Episodic memory appears to be negatively affected by both normal aging [3,4,5] and neuropathology [1]. Moreover, episodic memory impairment is an early clinical sign of typical Alzheimer’s disease [6] that precedes the decline in other cognitive domains [7,8]. The decline in episodic memory can be attributed to the inability to retrieve associations between cues and events [9]. Problems with episodic memory commonly serve as indicators of injury or disease of the brain. According to Deluca and Chiaravalloti, neuropsychological assessments of episodic memory often adhere to a two-step design: (1) Study phase: participants are presented with to-be-learned information (e.g., shopping list) and instructed to purposely memorize the material; and (2) Test phase: participants are asked to recall the previously learned information using free recall, cued recall, and recognition tasks [10]. For example, the California Verbal Learning Test, Second Edition (CVLT-II) was developed to assess a broad range of verbal episodic memory-related functions. Neuropsychologists working with patients who are experiencing progressive neurodegenerative diseases often administer the CVLT. Today, the CVLT is one of the most widely used assessments to evaluate memory [11,12].

### 1.1. Experimental Control and Ecological Validity

Like the CVLT-II, many traditional clinical and experimental tests of cognition in use today were developed to measure abstract cognitive constructs with as much task purity (experimental control) as possible. A difficulty in developing these memory tests is the tradeoff between ecological validity with experimental control [13]. Often neuropsychologists emphasize experimental control in their memory assessments given its importance for the focused and standardized manipulation of construct variables (e.g., episodic memory). Unfortunately, this emphasis on experimental control results in diminished ecological validity and a lower-dimensional representation of the patient’s episodic memory performance in everyday activities. Hence, traditional neuropsychological assessments emphasizing experimental control may have limited ecological validity [14,15,16,17]. While a construct-driven task like the CVLT is able to predict some features of a patient’s episodic memory, it may not be reflective of a patient’s everyday memory performance. Burgess et al. have argued for the development of neuropsychological tests that represent everyday “functions” [15]. This “function-led” approach starts with directly observable everyday behaviors and proceeds backward a given sequence of actions that leads that behavior in everyday functioning. Shifting from construct-driven to function-led tests will allow for a better representation of functional performance [15].

### 1.2. Virtual Environments for Function-Led Measures

Virtual reality offers a platform for a function-led measure that simulates daily activities in a standardized manner [18,19,20,21]. Virtual reality is an immersive environment that allows people to manipulate, visualize, and interact with computers in a complex manner, providing many new opportunities for clinical research and assessments [22]. Function-led virtual reality assessments may enhance ecological validity and better predict real-world functioning abilities [23,24]. This approach allows everyday behaviors and the ability to complete activities to be observed while implementing distractors. Utilizing the virtual environment and real-world distractors provides a potential for increasing ecological validity [25,26].

### 1.3. Virtual Reality-Based Assessment of Episodic Memory

Recent studies with non-clinical populations have emerged that explore the effects of aging on episodic memory using virtual environments. For example, Plancher and colleagues found that while recall of previously learned items remained intact there were age-related declines in spatiotemporal information retrieval [27]. Moreover, there are a growing number of studies comparing the episodic memory performance of younger and older adults in virtual reality-based shopping tasks with performance measures derived from the CVLT. Findings from these studies have revealed that virtual reality-based episodic memory assessments are consistent with traditional measures of the episodic memory construct [28,29,30,31].

### 1.4. The Current Study

Episodic memory is typically measured with list learning [1] and recognition [32] tasks like the California Verbal Learning Test (CVLT). A recent addition to episodic memory assessment is the Virtual Environment Grocery Store (VEGS). The VEGS is a virtual multiple errand platform that measures both episodic and prospective memory [28,31,33]. Participants are asked to carry out various day-to-day activities within the virtual environment to reflect their functional abilities. Like the CVLT-II, the VEGS uses list learning and recognition tasks to assess episodic memory [33]. Previous research investigated the construct validity of the VEGS via comparison with a traditional list-learning measure of episodic memory (i.e., the CVLT) and executive functioning (i.e., Delis–Kaplan Executive Function System Color-Word Interference Test; D-KEFS-CWIT) among young adults and healthy older adults [28]. This previous study is notable for not including everyday distractors in the virtual environment and the lack of a clinical sample.

In another study, Parsons and McMahan added various auditory and visual distractors to the VEGS [33]. Examples of auditory distractors include announcements given over the public-access system (e.g., clean-up needed on aisle seven; sales promotion announcements), smartphone alerts, laughter, coughing, and a baby crying. Visual distractors were also added: merchandise was dropped on the floor, there was increased clutter in the aisles, and several virtual humans were added [33]. An experiment involving a low distractor condition and another experiment involving a high distraction condition were conducted with two cohorts of healthy college-aged adults [33]. Performances on the VEGS memory tasks (low and high distraction conditions) and the traditional neuropsychological assessments of memory were positively correlated. The addition of distractors into the VEGS (high distraction condition) resulted in significant correlations with traditional measures of inhibitory control. Interestingly, for both studies, the recall was greater for VEGS than for CVLT items. However, the standard deviations for the VEGS items were notably larger than those for the CVLT. This is in contrast to a test–retest study by Weitzner et al., in which list learning (encoding) and list recall (retrieval) scores were both lower for the VEGS (high distraction condition) than they were for the CVLT [31].

The current study ran clinical and non-clinical participants in the high distraction paradigm. While previous studies used only a healthy older adult and younger adult population, the current study also administered the VEGS to older adults with neurocognitive impairments. The purposes of this study include:

1. Replication of prior construct validity results among a new sample of young adults and healthy older adults;
2. Extension of this work to a clinical sample of older adults with a neurocognitive diagnosis;
3. Comparison of CVLT-II and VEGS performance among these groups;
4. Validation of the independence of CVLT and VEGS episodic memory performance measures from executive functioning performance measures.

We hypothesized the following: (H_1_) that there would be moderate to high correlations between the VEGS measures of episodic memory and the analogous episodic memory measures on the CVLT-II. Considering the results of previous studies, we also hypothesized (H_2_) that, compared to the CVLT-II, participants (and particularly older adults and older adults with neurocognitive impairment) would recall significantly fewer items on the VEGS. We hypothesized (H_3_) that the VEGS episodic memory measures are independent from executive function measures in the younger and older adult population.

## 2. Materials and Methods

### 2.1. Participants

Participants (*N* = 164) consisted of a young adult and older adult age cohort; however, eight cases that represented univariate outliers were removed, leaving a final sample of *N* = 156. The young adult sample consisted of undergraduate students (*n* = 53; 64.15% female, 33.96% male, 1.9% no response; age 18–26, *M* = 19.11, *SD* = 1.79) enrolled in a psychology course at a public university in the southern U.S. This sample composed of all four class classification: Freshman (*n* = 39, 73.6%), Sophomore (*n* = 4, 7.5%), Junior (*n* = 7, 13.2%), and Senior (*n* = 3, 5.7%) with individuals completing at least a high school education. These students were recruited through the department’s SONA website, where students can volunteer to participate in research studies in exchange for course credit. Two groups of older adults were considered. The first group—the healthy older adult group (HOA)—consisted of community-dwelling older adults who agreed to participate in a research project (*n* = 85; 56.47% female, 38.82% male, 4.7% no response; age 55–89, *M* = 71.72, *SD* = 7.53). HOA participants indicated their highest level of education: Others (*n* = 4, 4.7%;, i.e., 10, 13, and 14 years), High school (*n* = 23, 27.1%), Associates degree (*n* = 12, 14.1%), Bachelor’s degree (*n* = 21, 24.7%), Masters degree (*n* = 14, 16.5%), Doctoral degree (*n* = 3, 3.5%), and missing (*n* = 8, 9.5%). The second group—the clinical older adult group (COA)—consisted of older adults who presented for a neuropsychological evaluation (*n* = 18; 38.88% female, 61.11% male, 1.9% no response; age 59–90, *M* = 78.17, *SD* = 8.52) at a university-affiliated neuropsychology clinic. COA participants indicated their highest level of education: Others (*n* = 3, 16.7%;, i.e., 9, 13, and 14 years), High school (*n* = 2, 11.1%), Associates degree (*n* = 2, 11.1%), Bachelor’s degree (*n* = 6, 33.3%), and Masters degree (*n* = 5, 27.8%). All of these adults were found to have a diagnosis of a mild (*n* = 13) or major neurocognitive impairment (*n* = 5) according to *DSM-5* criteria in a clinical evaluation supervised by a clinical neuropsychologist. Mild neurocognitive impairment included conditions such as mild cognitive impairment, unspecified minor neurocognitive disorder, and normal hydrocephalus pressure. Major neurocognitive impairments included major neurocognitive disorder due to Alzheimer’s disease, due to vascular disease, and unspecified. Participants were given a diagnosis after their performance in the neuropsychological evaluation and clinical patient and informant interviews as this study was a part of a larger clinical study.

Participant selection was based on individuals who were at least 18 years old. Individuals in the young adult group had to be within the age of 18 to 30 and had to be healthy with no reportable cognitive deficits. Individuals in the older adult group had to be within the age of 55 and older with either no neurocognitive diagnosis or one neurocognitive diagnosis. Participants who were under 18 years of age and did not complete the tasks were excluded from the study.

### 2.2. Measures

#### 2.2.1. California Verbal Learning Test—Second Edition

The California Verbal Learning Test—Second Edition (CVLT-II) measures verbal learning and memory [34]. The CVLT-II test also assesses verbal episodic memory, particularly the aspects of encoding, recall, and recognition [35]. During this test, participants are verbally presented with a list of sixteen categorized words where they must remember as many items as they can. During this test, participants are given five trials to freely recall the items back to the examiner. They are given both a short delay where they are read a second list of words and are asked to freely recall words from that second list. Immediately after the second list, they were asked to recall words from the first list that was read to them, both freely and when given verbal cues. Participants are given a long delay that lasts approximately 30 min. During that time, the test administrator would give the participant a nonverbal test. Following the delay, they are then asked to recall as many of the words as they can from the first list that was read to them, both freely and when given verbal cues. During this test, they also have a recognition portion where they must recognize which of the two words verbally presented to them was on the first list.

#### 2.2.2. Delis–Kaplan Executive Function System Color-Word Interference Test

The Delis–Kaplan Executive Function System (D-KEFS) Color-Word Interference Test (CWIT) is used to assess executive functioning [36]. D-KEFS CWIT consists of four conditions: (1) Color naming, (2) Word reading, (3) Inhibition, and (4) Inhibition/Switching. The color naming condition is the first condition, in which the participant is presented and asked to name the colored squares (i.e., red, blue, or green) as quickly and accurately as possible. The word reading condition is then presented in which the participant is presented and asked to read the words “red”, “blue”, and “green” printed in black ink as quickly and accurately as possible. The inhibition condition is the third condition, in which the participant is presented and asked to read the words “red”, “blue”, and “green” printed incongruently in red, blue, and green ink as quickly and accurately as possible. This third condition is known as the classic Stroop Task, where participants are required to name the ink color, instead of the color words. The inhibition/switching condition is the last condition, in which the participant is presented and asked to read the words “red”, “blue”, and “green” printed incongruently in red, blue, and green ink as quickly and accurately as possible (as in the third condition). Half of the words are within boxes, in which they are to read the word aloud and not name the ink color. Performance is measured by the time completed on each of the four conditions.

#### 2.2.3. Virtual Environment Grocery Store

**Figure 1:**
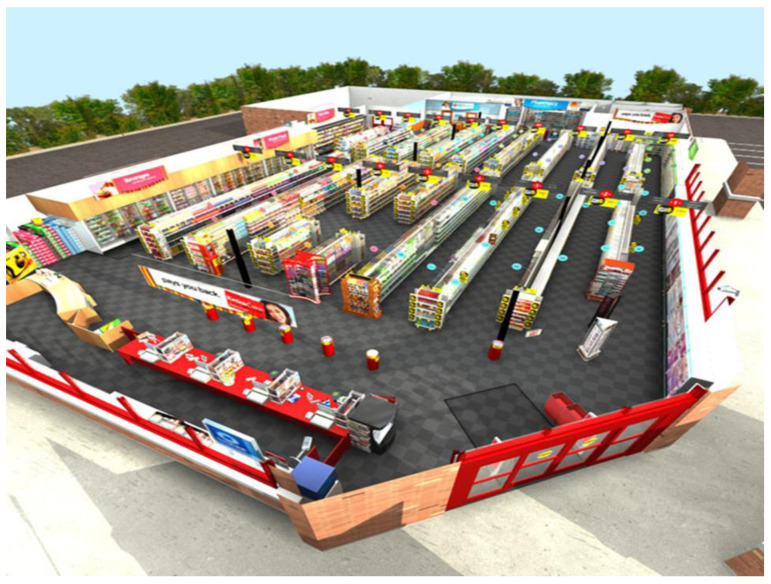
Virtual Environment Grocery Store.

**Figure 2:**
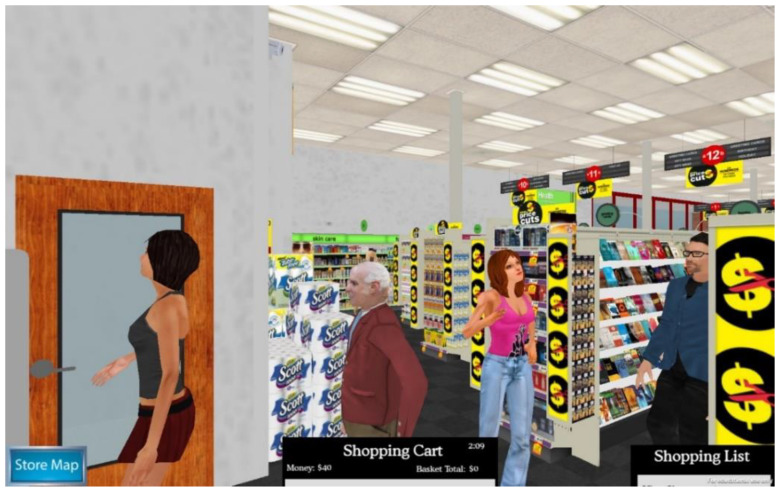
Everyday distractors in the Virtual Environment Grocery Store.

The Virtual Environment Grocery Store (VEGS; see Figure 1) is used to assess multiple neurocognitive measures including multitasking, prospective memory, and episodic memory in varying levels of environmental distraction [28,33]. Before entering the VR environment, participants are read sixteen items from a shopping list. Similar to the CVLT-II, they are exposed to five learning trials for encoding the shopping items. After completing the learning and immediate recall trials, participants are immersed in the virtual environment. Participants are instructed to drop off a prescription at the pharmacy and remember to listen for their prescription number (event-based prospective memory) as they pick up items on the shopping list (until their prescription is ready). They are also asked to remember to go to the coupon machine five minutes after dropping off their prescription (time-based prospective memory). After the immersion trial is over, participants are asked to recall (episodic memory recalled freely and with provided cues) as many of the items from the shopping list as they can. Participants are also given recognition trials. While immersed in the virtual environment, participants are exposed to several everyday environmental distractors. In addition to general ambient noise that one would find in an everyday real-world shopping environment, participants were exposed to various virtual human avatars (some avatars ambulating throughout the store, while others stood in place) that interacted with each other, spoke on virtual phones, or interacted with a crying baby avatar (see Figure 2). Furthermore, there were increased audio distractors: announcements are given over the public-access system, human laughter, coughing, baby crying; and various ring tones on cell phones. The episodic memory measures (i.e., learning, immediate, and delayed recall and recognition) were the primary measures used in this study. The shopping task measures were not the focus of this paper.

### 2.3. Procedure

This study was approved by the university’s committee for the protection of human subjects, and informed consent was obtained from all participants. All participants were tested at an outpatient clinic/lab space in which participants completed a larger test battery. The order of tests presented was counterbalanced to guard against order effects.

### 2.4. Data Analysis

All analyses were conducted in SPSS version 26 (IBM SPSS Statistics). As noted earlier, eight cases were univariate outliers and were removed prior to analysis. Univariate outliers were detected through SPSS boxplots and with an absolute value of 3.29 [37]. Two cases of multivariate outliers were detected through Mahalanobis distance; however, they were not removed as they did not have undue influence on the distribution. Homogeneity of covariance was tested through Box’s M Test with a significant result, suggesting that the assumption is not met. Therefore, Pillai’s Trace statistics were reported to provide a more robust result [38].

## 3. Results

One-way factorial multiple analysis of covariance (MANOVA) compared the three groups (i.e., young adults, older adults without a neurocognitive disorder, and older adults with a neurocognitive disorder) on the two different tests (i.e., CVLT-II and VEGS) for immediate recall, delayed free recall, delayed cued recall, and recognition. Group membership had a large multivariate effect, Pillai’s Trace = 0.56, *F*(8, 284) = 13.71, *p* < 0.001, η*_p_*^2^ = 0.27. A multivariate effect was found for test, Pillai’s Trace = 0.07, *F*(4, 141) = 2.60, *p* = 0.039, η*_p_*^2^ = 0.07. There was also a significant multivariate group x test interaction, Pillai’s Trace = 0.19, *F*(8, 284) = 3.79, *p* < 0.001, η*_p_*^2^ = 0.10. However, no significant effects were found for education (Pillai’s Trace = 0.02, *F*(4, 141) = 0.70, *p* = 0.59, η*_p_*^2^ = 0.02) and test interaction x years of education (Pillai’s Trace = 0.04, *F*(4, 141) = 1.49, *p* = 0.21, η*_p_*^2^ = 0.04).

Univariate within-subjects differences were found between performance on the VEGS immediate recall and CVLT-II immediate recall, *F*(1, 144) = 9.40, *p* = 0.003, η*_p_*^2^ = 0.06. However, there was no univariate difference in long delay free recall (*p* = 0.071), long delay cued recall (*p* = 0.070), and recognition (*p* = 0.29). No significant interaction between groups and tests was found for delayed free recall and delayed cued recall. There was a significant interaction between groups and immediate recall, *F*(2, 144) = 3.08, *p* = 0.05, η*_p_*^2^ = 0.04. There was also a significant interaction between groups and recognition, *F*(2, 144) = 6.10, *p* = 0.003, η*_p_*^2^ = 0.08. Univariate between-subjects (i.e., group) differences were found on VEGS variables, and these are displayed in Table 1. Post hoc analysis with Tukey’s HSD found that all group differences (i.e., immediate recall, long delay free recall, and long delay cued recall) were significant at *p* ≤ 0.001. There was a significant difference between young adults and older adults without a neurocognitive diagnosis on VEGS recognition (*p* = 0.010). See Table 2.

Construct validity results for the young adult, older adults without a neurocognitive diagnosis, and older adults with a neurocognitive diagnosis can be found in Table 3. Younger-aged group’s CVLT-II and VEGS memory performance did not correlate with traditional executive functioning measures (i.e., D-KEFS CWIT Inhibition and Inhibition/Switching). Similar results were also observed among older adults with a neurocognitive diagnosis. Their CVLT-II and VEGS memory performance, generally, did not correlate with D-KEFS CWIT. Results indicated that VEGS recognition and D-KEFS CWIT Inhibition/Switching, however, were correlated with this older adult group. Additionally, the result of CVLT-II and VEGS performance among older adults without a neurocognitive diagnosis indicated correlations with D-KEFS CWIT measures.

## 4. Discussion

The purposes of this study include (1) Replication of prior construct validity results among a new sample of young adults and healthy older adults; (2) Extension of this work to a clinical sample of older adults with a neurocognitive diagnosis; (3) Comparison of CVLT-II and VEGS performance among these groups; and (4) Validation of the independence of CVLT and VEGS episodic memory performance measures from executive functioning performance measures. Results were consistent with H_1_ in that the relationship between the VEGS measures and the analogous measures on the CVLT-II were highly correlated on all variables. Furthermore, the results from this study supported H_2_, showing that compared to the CVLT-II, participants (particularly older adults with neurocognitive impairment) recalled fewer items on the VEGS. Finally, H_3_ was supported in that the VEGS episodic memory measures were found to be different from executive function measures in the younger and older adult populations.

### 4.1. Discussion of Hypothesis

#### 4.1.1. Hypothesis 1: Convergent Validity on Virtual and Traditional Measures of Episodic Memory

Results were consistent with H_1_ in that the relationship between the VEGS measures and the analogous measures on the CVLT-II were highly correlated on all variables. These results are consistent with aging previous studies that compared the CVLT and virtual reality-based measures of episodic memory [28,29,30,31].

#### 4.1.2. Hypothesis 2: Word Recall on Virtual and Traditional Measures

Results from this study supported H_2_ showing that compared to the CVLT-II, participants (particularly older adults with neurocognitive impairment) recalled fewer items on the VEGS. While these results are consistent with recent test–retest results that found both list learning (encoding) and list recall to be lower for the VEGS (high distraction condition) than they were for the CVLT, they appear to be in contrast with early findings that participants did better on VEGS recall in both low distraction and high distraction conditions of the VEGS [28,31,33]. See Table A1. There are two likely interpretations for this result. First, there was considerably more variability in scores for both the CVLT-II and VEGS scores in this study compared to previous work. Moreover, the VEGS was administered with several everyday environmental distractors present (high distraction condition), which could have resulted in greater variability in the items recalled from the VEGS in the delayed recall and recognition. This result is consistent with the findings (time 1 and time 2) of Weitzner et al. and is consistent with the notion that distractors may have the greatest harmful effects on the recall of older adults compared to young adults [33,39,40]. Previous research has shown that the presence of distractors negatively impacts recall among both young adults and older adults; however, distractors may be more disruptive to recall among older adults [41]; these results from Parson and McMahan are displayed in Table A2 [33].

It is important to note that the differences between the CVLT-II and VEGS occurred on immediate recall, prior to the presence of distractors. Therefore, another plausible explanation for these results is the word length effect [42]. The word length effect [42,43,44] refers to the fact that lists of short words are recalled better than lists of long words. While the original work on the word length effect was done in the context of short-term memory and working memory, the effect is also found in free recall experiments [45]. The average number of syllables for the CVLT-II words is 2.37, whereas for the VEGS it is 3.67.

#### 4.1.3. Hypothesis 3: Divergent Validity for Virtual Reality Measures of Episodic Memory

Results supported H_3_ in that both CVLT-II and VEGS episodic memory measures were not correlated with traditional executive functioning measures among younger adults. This result is consistent with previous studies that investigated the construct validity of both traditional and VEGS memory performance with traditional executive functioning measures [28,33]. Older adults without a neurocognitive diagnosis indicated that both traditional and VEGS episodic memory performance involved some levels of executive functioning. This is consistent with previous findings that executive functioning can play a role in episodic memory performance in healthy older adults [33,46]. Notably, the results of older adults with a neurocognitive diagnosis indicated that both traditional and virtual memory assessments were generally independent of traditional executive functioning measures. The weak to moderate correlations between inhibition and episodic memory in healthy older adults may reflect the ability of executive function to suppress irrelevant information [47].

### 4.2. Limitations

It should be noted that the findings of the present study should be understood in the context of some limitations. First, the samples were fairly small and not necessarily representative of their respective age cohorts. The samples were also not fully equivalent; they were recruited with different approaches and not matched on factors like income. A further issue is that while the current study counterbalanced the order of presentation, future studies should examine potential mediating effects of conflict processing differences between low and high distractors. Moreover, effects may be due to social versus neutral distractors. A further issue of exploration is the issue of the list learning and the word-length effect. Future studies should investigate the learning slope for both CVLT and VEGS list learnings across learning trials. This may help in disambiguating findings related to encoding and recall. Future studies could include a larger clinical sample and a comparison of performance in VEGS conditions with and without distractors. Finally, future studies should investigate the performance of clinical cohorts on the prospective memory and everyday shopping tasks found in the VEGS.

## 5. Conclusions

Overall, this study adds to the literature supporting the construct and divergent validity of the VEGS through its correlations with analogous variables on the CVLT-II. Moreover, it enhances our understanding of the potential utility, as well as psychometric properties, of the VEGS for episodic memory measures across the lifespan. Relatedly, these findings offer growing support for the use of virtual reality-based neuropsychological measures of episodic memory in aging clinical populations.

## Figures and Tables

**Table 1:** Univariate group differences between subtests and descriptive statistics on the VEGS variables (*N* = 156) with education as a covariate.

				YA (*n* = 53)	HOA (*n* = 85)	COA (*n* = 18)
	F(2144)	*p*	η_p_^2^	M (SD)	M (SD)	M (SD)
VEGS Immediate Recall	72.86	<0.001	0.50	51.89 (8.69)	43.66 (12.02)	19.33 (8.24)
VEGS Long Delayed Free Recall	57.65	<0.001	0.45	10.89 (2.83)	9.20 (3.47)	3.44 (3.45)
VEGS Long Delayed Cued Recall	64.05	<0.001	0.47	10.72 (2.51)	9.61 (3.28)	3.11 (2.91)
VEGS Forced-Choice Recognition	22.14	<0.001	0.24	15.85 (0.41)	15.27 (1.33)	12.44 (3.50)

**Table 2:** Bivariate correlations between subtests with descriptive statistics for all variables of interest (*N* = 156, all correlations significant at *p* < 0.001).

	1	2	3	4	5	6	7	8
1. CVLT-II Immediate Recall	-	0.87	0.87	0.58	0.80	0.71	0.70	0.61
2. CVLT-II Long Delayed Free Recall		-	0.93	0.57	0.78	0.70	0.71	0.62
3. CVLT-II Long Delayed Cued Recall			-	0.61	0.78	0.67	0.69	0.64
4. CVLT-II Forced-Choice Recognition				-	0.48	0.38	0.40	0.49
5. VEGS Immediate Recall					-	0.83	0.82	0.61
6. VEGS Long Delayed Free Recall						-	0.78	0.58
7. VEGS Long Delayed Cued Recall							-	0.63
8. VEGS Forced-Choice Recognition								-
M	48.24	10.24	10.99	14.53	43.65	9.11	9.24	15.14
SD	13.17	4.22	3.78	2.07	14.25	3.92	3.75	1.84

**Table 3:** Bivariate correlations between variables with descriptive statistics for convergent and divergent validity results for the group memberships (*N* = 156).

	YA (*n* = 53)				HOA (*n* = 85)				COA (*n* = 18)			
Variable	D-KEFS Color Naming	D-KEFS Word Reading	D-KEFS Inhibition	D-KEFS Inhibition/Switching	D-KEFS Color Naming	D-KEFS Word Reading	D-KEFS Inhibition	D-KEFS Inhibition/Switching	D-KEFS Color Naming	D-KEFS Word Reading	D-KEFS Inhibition	D-KEFS Inhibition/Switching
CVLT-II												
Immediate Recall	0.2	−0.13	−0.04	0.003	−0.41 ***	−0.2	−0.46 ***	−0.21	−0.19	−0.05	0.11	−0.37
Long Delayed Free Recall	0.01	0.027	−0.12	−0.07	−0.36 **	−0.19	0.40 ***	−0.17	−0.13	−0.04	0.1	−0.29
Long Delayed Cued Recall	0.13	0.06	−0.02	0.09	−0.36 **	−0.2	−0.38 ***	−0.17	−0.16	0.002	0.1	−0.33
Forced−Choice Recognition	−0.15	−0.13	0.07	0.03	−0.33 **	−0.25 *	−0.24 *	−0.08	−0.02	0.16	−0.05	−0.4
VEGS												
Immediate Recall	0.30 *	−0.29 *	−0.21	−0.17	−0.46 **	−0.35 **	−0.48 ***	−0.43 ***	−0.26	−0.23	−0.04	−0.21
Long Delayed Free Recall	−0.07	−0.13	0.04	−0.1	−0.38 **	−0.24 **	0.43 ***	−0.32 **	−0.17	−0.10	−0.002	−0.21
Long Delayed Cued Recall	−0.17	−0.16	−0.17	−0.22	−0.35 **	−0.31 **	−0.45 ***	−0.37 **	−0.32	−0.074	−0.1	−0.34
Forced-Choice Recognition	0.11	0.34	0.1	−0.11	−0.30 **	−0.25 **	0.28 **	−0.2	−0.003	0.19	0.45	−0.56 *
M	26.79	20.06	45.79	54.47	32.89	24.28	70.69	76.26	39.51	26.87	96.72	95.44
SD	4.78	3.42	9.63	12.28	6.61	4.31	21.02	18.53	10.36	6.25	40.25	33.05

* *p* < 0.05. ** *p* < 0.01. *** *p* < 0.001.

## Data Availability

Data is available upon request.

## Appendix A

**Table A1:** Comparison of group performance on neuropsychological measures [28].

	Parsons & Barnett, 2017		Present Study		
Variable	Young Adult M (SD)	Older Adult M (SD)	Young Adult M (SD)	Healthy Older Adult M (SD)	Clinical Older Adult M (SD)
CVLT-II Long Delayed Free Recall	7.40 (1.40)	6.40 (2.20)	12.58 (2.35)	10.22 (3.51)	3.39 (4.18)
CVLT-II Long Delayed Cued Recall	7.60 (1.50)	6.70 (2.00)	13.23 (1.95)	10.88 (3.18)	4.94 (3.69)
CVLT-II Forced Choice Recognition	-	-	15.26 (1.18)	14.35 (2.18)	13.22 (2.82)
VEGS Long Delayed Free Recall	9.67 (1.94)	6.97 (3.30)	10.89 (2.83)	9.20 (3.47)	3.44 (3.45)
VEGS Long Delayed Cued Recall	9.42 (1.91)	7.07 (3.32)	10.72 (2.51)	9.61 (3.28)	3.11 (2.91)
VEGS Forced Choice Recognition	----------	----------	15.85 (0.41)	15.27 (1.33)	12.44 (3.50)

**Table A2:** Comparison of distractor performance on neuropsychological measures [33].

	Distractors		Non- Distractors	
Variable	M	SD	M	SD
CVLT-II Long Delayed Free Recall	7.34	1.24	7.24	1.45
CVLT-II Long Delayed Cued Recall	7.52	1.31	7.45	1.58
VEGS Long Delayed Free Recall	9.08	2.35	9.67	2.00
VEGS Long Delayed Cued Recall	9.00	2.71	9.45	2.03
